# The Density of Recombination-Associated Genomic Features Does Not Generally Explain the Broad-Scale Crossover Patterns in Chicken and Guinea Fowl

**DOI:** 10.3390/ani15121759

**Published:** 2025-06-14

**Authors:** Luis F. Rossi, María Inés Pigozzi

**Affiliations:** Instituto de Investigaciones Biomédicas (INBIOMED), Facultad de Medicina, Universidad de Buenos Aires, CONICET, Buenos Aires C1121ABG, Argentina

**Keywords:** meiotic recombination, GC content, CpG islands, meiotic chromosomes, cytogenomics

## Abstract

Meiotic recombination is essential for chromosomal segregation and facilitates the exchange between homologs, which leads to the transmission of new combinations of linked alleles to the progeny. The eukaryotic meiotic machinery is generally highly conserved, but the frequency of crossover occurrence can vary dramatically across species and populations, between individuals, and across sexes. The chicken and the guinea fowl exhibit interspecific variation in the distribution of crossovers along their largest chromosomes. In many organisms, an association has been observed between the preferred crossover location and certain sequence parameters, such as high GC content, CpG islands, or gene promoters. Here, we compared the distribution of these genomic parameters with the recombination landscape, represented by MLH1 focus frequencies, in the two birds. We found an association between GC content density and recombination in the chicken, but the remaining parameters showed weak or no association with recombination, especially in the guinea fowl. We conclude that despite the different broad-scale crossover distribution, the investigated genomic parameters remained remarkably similar in these two species. We suggest that the density of these genomic features is more likely related to microscale variations in recombination rates, such as those determined by open chromatin configurations.

## Simple Summary

In spite of the evolutionary conservation of the meiotic machinery, the frequency and distribution of the crossover events can display ample differences between species or individuals. The occurrence of crossover events has been associated with certain sequence features in the genome, such as the proportion of GC content. Here, we investigated the distribution of certain genomic features in two birds that have striking differences in their recombination landscapes: the chicken and the guinea fowl. If the investigated genomic features are related to the differences in recombination rates at a broad scale, then the relative density of these features should differ across the genome of the investigated species.

## 1. Introduction

Meiotic crossovers (a form of meiotic recombination) break associations between alleles at linked loci, resulting in new haplotypes. This affects how selection acts during molecular evolution, as non-recombining genomic regions of sexually reproducing organisms deteriorate by the accumulation of harmful mutations [1,2]. Also, the correct number and placement of meiotic crossovers have a vital role in ensuring faithful segregation, with failures often resulting in aneuploidy and infertility. Understanding how the frequency and distribution of crossovers are determined is crucial because these parameters influence levels of genetic variability, the efficacy of selection, and the effectiveness of association and linkage mapping studies [3,4].

The frequency and the distribution of crossover events in a genome can be investigated at different resolutions, from a few kilobases to several megabases. High-resolution recombination maps can be obtained by indirect inference of crossover events from haplotype shifts across generations using DNA sequence information. These methods may include population or pedigree analyses or have a gamete-based approach [5,6,7,8,9]. In spite of their kb level resolution, population-based estimates of recombination rates have certain limitations, such as the fact that genetic variation is necessary to detect historical recombination events, and that linkage disequilibrium (LD) is affected by the effective population size of a population, which is influenced by the population’s demographic history such as bottlenecks, genetic drift or selection [9]. Another drawback of estimating recombination rates from genomic data is that the results are highly dependent on sequencing coverage of the genome, making it difficult to draw conclusions for comparative analysis.

Meiotic recombination rates can also be studied by tracking the physical placement of crossovers along chromosomes using cytological methods. The immunodetection of MLH1, a mismatch repair protein that localizes to late recombination nodules at the pachytene stage of meiosis, yields a precise assessment of the number and distribution of crossover events along chromosomes [10,11,12,13,14,15,16]. Although this cytological approach only provides a relatively coarse (Mb) resolution of the variation in recombination rates across the genome, it can directly access information that is frequently concealed or inferred indirectly from the genomic methods, enabling direct comparisons of recombination rates between different studies [17,18]. Among birds, MLH1-focus mapping has been employed in fewer than 20 species [19], but some general features regarding crossover distribution along macrochromosomes can be pointed out. In most species, crossovers are more or less homogeneously distributed in the six to eight largest chromosomes (macrochromosomes), very often with more than two crossovers per chromosome arm in the three longest pairs [20,21]. In other species, however, crossovers are localized towards the macrochromosome ends, with large recombination “desserts” in the central regions [15,22]. Comparative analysis throughout the avian phylogeny shows that broad-scale recombination patterns in the macrochromosomes shifted from multiple, less localized crossovers to crossover localization at different moments of avian evolution [22]. The chicken and the guinea fowl are representative of these two different patterns, and despite their divergence about 46 Mya, they share a high degree of chromosomal similarity with a low frequency of interchromosomal rearrangements [23]. In the chicken, focus frequencies are higher at the chromosome ends, but they are relatively homogeneous in the center. Instead, foci are strongly biased towards ends in guinea fowl, with nearly 80% of crossovers mapping within a fifth of the macrochromosome lengths and a sharp decline in their frequency in the mid-chromosomal regions [22].

The analysis of recombination rates and patterns across species and populations, between individuals, and across sexes show differences that are apparently associated with a combination of interacting environmental, epigenetic, and genetic factors [24,25,26]. Along with these factors, certain genomic parameters such as GC content, CpG islands, and promoters (genes) were found to be related to higher recombination rates at a few kilobase resolution levels in many organisms, including birds [4,27,28,29]. If recombination rates at a broad scale also lead (cause) or allow differences such as in GC content, then the density of the mentioned genomic parameters should differ in species with divergent crossover patterns. To test this, we compared the distribution of GC content, CpG islands, and coding regions (genes) in the chicken and the guinea fowl. First, we converted the MLH1 frequencies into cM/Mb recombination maps in each species and compared the recombination rates in homologous segments of the macrochromosomes. Then, by dividing the chromosome’s physical length into intervals equivalent to 2.5 Mb, we calculated the GC content, CpG island, and gene density along homologous segments and compared them between species. We found that these recombination-associated parameters cannot explain completely the differences in recombination distribution found at large scales in the chicken and the Guinea fowl. This study contributes to the broader understanding of avian genomics and highlights the remarkable differences in genetic recombination of the chicken and guinea fowl.

## 2. Materials and Methods

### 2.1. Chicken and Guinea Fowl Recombination Maps

Chicken and guinea fowl karyotypes are typical of avian genomes, with a few macrochromosomes and a much larger set of smaller chromosomes (microchromosomes). Their diploid numbers differ by one pair, being 2n = 78 in the chicken and 2n = 76 in the guinea fowl [22]. Cytogenetic and genomic analyses show strong genome collinearity between the species, with few interchromosomal rearrangements: guinea fowl chromosome 4 (NME4) corresponds to chicken chromosome 9 (GGA9) and GGA4q; NME5 to GGA6 and GGA7; NME6 to GGA5 and NME7 to GGA8 [23,30]. The karyotype comparison of chicken and guinea fowl macrochromosomes is shown in Figure S1 to make the graphical representations of the data analysis easier to understand. The crossover distributions on the six largest chromosomes of the Guinea fowl and the seven largest chromosomes of the chicken were obtained by immunocytological detection of the protein MLH1 during late pachytene in 138 chicken oocytes and 133 oocytes from the guinea fowl. This experimental data is part of previous work in our laboratory at https://doi.org/10.1371/journal.pone.0240245.s001 (accessed on 30 March 2024) [22].

### 2.2. Conversion of Physical Distances Along Chromosomes into Genomic Distances

To compare the distribution of crossovers and genomic features in matching regions along chromosomes, it is required to convert the physical distances, measured in micrometers or percentages, into genomic distances in base pairs. The use of pachytene chromosomes is particularly advantageous in this regard because, at this stage of meiosis, genomic distance is proportional to the physical distance along the chromosome axes. This is so because the DNA packing ratio along the chromosomes is relatively constant, as demonstrated in a large variety of organisms, including birds [31,32,33]. Therefore, the segments’ fractions of total physical axis length are approximately equal to their fractions of total genomic length. We divided the total assembly length of each chromosome in the chicken and guinea fowl assembly into 2.5 Mb segments. This interval size was selected because it corresponds to a physical distance that can be resolved by the measuring device on digitized images of the synaptonemal complex in bird oocytes [34]. For example, chromosome 1 has 194.4 Mb in the guinea fowl genome assembly (NuMel1.0), and the average length of SC1 is 24.5 μm [22]; therefore, there are 78 intervals of 2.5 Mb, equivalent to approximately 0.3 μm each. Given that the macrochromosomes of chicken and guinea fowl are comparable in size [23,35], there is little to no difference in the number of intervals per chromosome or chromosome arm (Table S1).

### 2.3. Recombination Maps Along Macrobivalents

Recombination rates for each interval were obtained from the MLH1 focus counts in the six or seven largest macrobivalentes of both species. Focus frequencies were converted to centimorgans (cM) by multiplying the number of foci in each interval by 50 and then dividing by the total number of SCs observed. This genetic length divided by the interval size in Mb gives the recombination rate in cM/Mb for each interval [14,31,32]. Comparisons between species were carried out at the chromosome level, taking into account the sequence homology between these species as established by cytogenetic and genomic studies (Figure S1). The short arms of GGA4 and NME4 were formed by translocations of different microchromosomes in each lineage [30], so our comparisons were limited to the long arm.

### 2.4. Maps of Sequence Features at the Chromosome Level

The GC content and the average densities of CpG islands (CGI) and genes were obtained from the genome assemblies of each species available at https://www.ncbi.nlm.nih.gov/genome/ (accessed in 30 March 2024). The GC levels were determined by scanning each macrochromosome sequence using EMBOSS Isochore (http://emboss.open-bio.org/ (accessed in 30 March 2024)) with the window size set to match the number of bins in the recombination histogram of each chromosome. The output file generated by the application was used to plot the GC levels in GraphPad Prism 8.0.1. The CpG island counts on each chromosome were calculated using the hgTables tool from the UCSC genome browser filtered as regions of DNA of length greater than 200 bp with a GC content of 50% or greater and observed CpG/expected CpG of 0.6 [36]. In both species, the gene start positions on each macrochromosome were also obtained using the hgTables tool. Then, we calculated the number of genes in each 2.5 Mb interval. In the graphs, the gene densities are expressed as the percentage of the total number of genes in a linkage group in a given interval. Correlations were established using Spearman’s correlation test in GraphPad Prism 8.0.

## 3. Results

**Figure 1:**
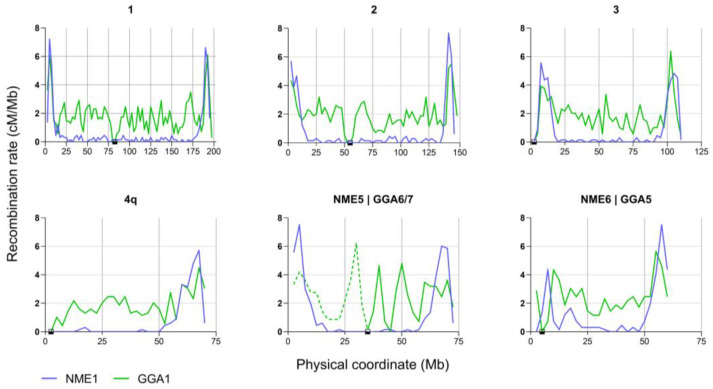
Recombination rates along the macrochromosomes of the chicken (GGA) and the guinea fowl (NME). The rates are expressed in cM/Mb, and each chromosome is divided into 2.5 Mb intervals. In each graph, the tip of the short arm is at 0 on the X-axis. NME5 corresponds to the fused chromosomes 6 and 7 of the chicken; GGA6 is represented as a dashed line because the physical coordinates were inverted to match the centromere-telomere orientation in both species. The squares on the X-axis mark the position of the centromeres.

The linear association between the synaptonemal complex length and the DNA content observed in birds enables the comparison of recombination rates obtained by physical crossover mapping (MLH1 foci) with the distribution of genomic sequences in the same chromosome segments (see Methods). To test if sequence features such as the GC content, CpG islands, and coding sequences are associated with the recombination differences observed at a broad scale in the chicken and the guinea fowl, we followed a multistep approach. First, we compared the recombination rates at equivalent chromosomes or chromosome segments divided into 2.5 Mb intervals (Figure 1 and Figure S2). The recombination rates in all the intervals ranged from 0 to 7.5 (average 2.0 ± 1.2) in chicken and from 0 to 7.7 (average 0.84 ± 1.7) in the guinea fowl. In both species, the recombination rates are high near the ends of the macrochromosomes, but in the mid regions, there are substantial differences, with a flatter profile in the guinea fowl. Steep declines are seen in recombination rates at the very ends of chromosomes. The regions with greater recombination levels are actually sub-terminal and span 5 to 7 Mb in both species.

**Figure 2:**
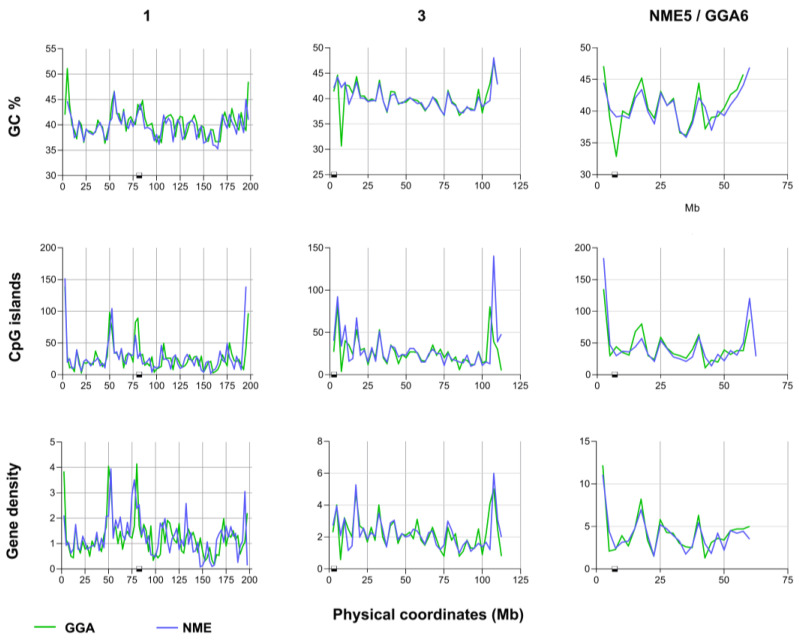
Genomic parameters densities in the chicken and the guinea fowl. The graphs show the percentage of GC content, numbers of CpG islands, and gene density in 2.5 Mb intervals along three representative macrochromosomes. The graphs for the other macrochromosomes can be found in the Supplementary Material Figures S2–S4. The squares on the X-axis mark the position of the centromeres.

Profiles for GC content and CpG islands are very similar in the chicken and the guinea fowl, especially in those chromosomes that are not rearranged between species (Figure 2 and Figure S2). Occasional exceptions include centromeric and pericentromeric regions that might respond to the presence of heterochromatin and its tendency to organize differently throughout the SC. We also examined the relationship between gene density and the recombination rates because an association has been observed between functional sequences (e.g., gene promoters) and COs in birds [6]. The species under study here have strikingly similar gene density profiles, with comparable peaks and valleys along most of the chromosome lengths (Figure 2 and Figure S4). It is possible that the size of the intervals in our study may conceal differences in these parameters between the two species. These differences would be apparent only at the microscale level, for example, if transcription start sites were examined.

If divergent densities of the quantities measured by these parameters are related to the species differences in recombination rates, then these distributions should differ in the two species because of the reduced recombination toward the chromosome centers in the guinea fowl and the lack of this reduction in the chicken. Instead, we observed that the landscapes of the investigated parameters are similar in both species, casting doubt on the hypothesis.

**Figure 3:**
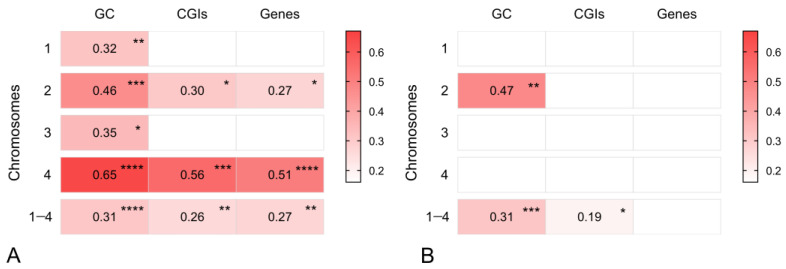
Association between recombination rates and genomic features calculated in 2.5 Mb windows. (**A**) Chicken. (**B**) Guinea fowl. Only the significant correlations are presented with their corresponding Spearman’s coefficients. The asterisks represent the *p*-value. The color intensity indicates the correlation strength. The complete data of correlation coefficients and their corresponding *p*-values can be found in the Supplementary Material Table S2.

In order to formally quantify species-specific associations between the genomic parameters and recombination rates, we tested the correlations between each parameter distribution and the recombination rates, both per chromosome, and gathered data for chromosomes 1 to 4. In the guinea fowl, segments with no recombination were excluded from the analysis to avoid the effects of data zero inflation because there is hardly any variation in the recombination rate in the central regions of chromosomes. In the chicken, most genomic features showed significant positive correlations, even though the correlation coefficients were not large, with 0.65 for the most associated parameter (Figure 3A). In the guinea fowl, only GC content in chromosome 2 and GC and CGI showed positive correlations with recombination rates when data from all chromosomes were pooled (Figure 3B). Given that most parameters showed no correlation or low significance in this species (Table S2), we conclude that the few positive correlations reflect stochastic noise rather than biological signals. Altogether, these results indicate that the differences in the broad-scale recombination patterns in the chicken and the guinea are only weakly related to the genomic parameters investigated here.

## 4. Discussion

In the present study, we investigated if the remarkably different crossover patterns in the chicken and guinea fowl macrochromosomes at a broad scale are related to the distribution of recombination-associated genomic sequences as observed at the fine-scale level. We found little evidence for a correlation between the investigated parameters and the recombination rates. Only the GC content showed a positive correlation with recombination rates in all the bivalents tested in the chicken, with substantial interchromosomal variations. This is not surprising since the correlation between GC content and recombination levels is variable between linkage groups in birds and other organisms [37]. An interchromosomal variation has also been observed at a fine-scale level in organisms with advanced recombination data. In the yeast *Saccharomyces cerevisiae*, the GC content is correlated with recombination rates in some chromosomes but not in others, suggesting that recombination is determined either by the GC content or by a third parameter, also affecting the GC content [38]. As a result, one could anticipate differences in the correlation coefficients similar to those seen in the chicken bivalents. In the guinea fowl, the association between GC content and crossover distribution is less consistent since only chromosome 2 shows a statistically significant correlation. This isolated significant value could reflect such interchromosomal variations, but it cannot be ruled out that it is due to random noise and lacks biological significance.

Unlike GC content values that were associated with recombination rates, at least in the chicken, CGIs and gene distributions showed inconsistent relationships with recombination rates. CGI and gene densities are correlated with recombination rates in some bivalents and not in others in the chicken, while they are not significantly related in the guinea fowl (Figure 3). The interchromosomal variations in CGIs vs. recombination rate association observed in the chicken might represent true biological signals because CGIs are not always associated with high recombination levels at kb intervals. Recently, only a weak association between CGI frequencies and recombination was reported in two species of passerine birds: the Eurasian blackcap and the garden warbler [39]. In these species, contradictory patterns were observed for some chromosomes within regions of high recombination and low CGIs (or the opposite). It was proposed that the lack of association of both parameters may be due to different methylation states in the various genomic regions. CGIs are usually associated with DNA hypomethylation at gene promoters, open chromatin state, and highly recombining regions. However, there are many hypermethylated CGIs located in intergenic DNA outside coding sequences [40], and in this case, recombination will be suppressed. These results support the idea that variations in recombination frequencies are not exclusively shaped by the location of functional elements but may also be influenced by species-specific methylation status.

Based on our results, we conclude that the distribution of genomic features associated with recombination at a fine scale does not account generally for the recombination patterns at a broad scale, especially in the guinea fowl. In birds, evidence from genetic linkage maps and cytogenetic crossover maps shows a tendency in crossover distribution toward the macrochromosome ends disregarding the presence of crossover polarization as observed in the guinea fowl and certain passerines [41,42,43]. The process of reciprocal recombination from its beginning through DNA double-strand breaks (DSBs) to the resolution of crossover events has not been revealed in detail among birds. However, the core meiotic processes are evolutionarily conserved in a wide range of organisms, from yeast to higher plants and animals [44], and therefore, it is plausible that the global regulation of crossover landscape in birds has common features with those observed in other organisms. The regulation of crossover distribution at the chromosome level has been examined in more detail in certain plants, like barley and wheat, in the domestic mouse and also in human oocytes and spermatocytes [45]. In these organisms, initial crossover events, such as DSBs and earlier recombination intermediates, occur regularly along the lengths of the chromosomes, while crossovers exhibit more localized distributions. This pattern corresponds to a temporal program in which DNA replication, early events of recombination, and SC formation all occur first near chromosome ends and then progress through middle regions [46]. Crossovers then occur differentially in the earlier-progressing regions, with recombination in middle regions presumably biased to give no crossovers. We suggest that the presence of higher crossover rates at the ends of avian macrochromosomes, with or without strong polarization, points to the existence of a conserved meiotic mechanism leading to preferred crossover designation at opposite ends of macrochromosomes. Initiation of synapsis at terminal regions of macrobivalents is documented in male and female birds [47,48] supporting a connection between early synaptonemal complex formation and preferential crossover designation at chromosome ends.

The variation in the number of crossovers at intermediate positions of the chromosome arms can be related to different modulations of the length of chromosome axes. There is growing evidence that the length of the chromosomal axes controls the amount and location of crossover events, with the total level of crossovers being proportional to the axis length [49,50]. It is interesting that the total synaptonemal complex set is shorter in the guinea fowl, and the total number of foci is lower compared to the chicken [22], as expected if the axis length is involved in the total amount of crossover events. In order to gain a better understanding of the factors governing the crossover patterns in birds, it is necessary to examine the molecular events involved in recombination complexes at the DNA level as well as the axis-associated elements of recombination that modulate crossover frequency.

## 5. Conclusions

We conclude that the differences in broad-scale crossover patterns observed in the chicken and the guinea fowl are not related to different distributions of recombination-associated sequences. Among the sequence parameters investigated here, only the GC content showed a positive relationship with recombination rates in the chicken, in agreement with the association previously reported at the fine (kb) level. The density of CGIs and gene promoters does not account for crossover frequencies at the Mb scale, suggesting that DNA modifications involved in open chromatin configurations must act coordinately with chromosome-scale factors, such as crossover interference, to promote the initiation of crossing over and to determine species-specific crossover patterns.

## Data Availability

All data are reported in this manuscript and its Supplementary Material.

## Supplementary Materials

The following supporting information can be downloaded at: https://www.mdpi.com/article/10.3390/ani15121759/s1, Figure S1: Comparative ideograms of chicken and guinea fowl 10 largest chromosomes; Table S1: Number of chromosome intervals per chromosome; Table S2: Association between sequence parameters and recombination rates; Figure S2: GC content; Figure S3: CGIs; Figure S4: Gene density.

## Abbreviations

The following abbreviations are used in this manuscript:

LD	Linkage disequilibrium
CGI	CpG islands
GGA	Gallus Gallus (chicken)
NME	Numida meleagris (guinea fowl)
