# Supplementary material for: The Density of Recombination-Associated Genomic Features Does Not Generally Explain the Broad-Scale Crossover Patterns in Chicken and Guinea Fowl
Source: Animals (Basel). 2025 Jun 14;15(12):1759. doi: 10.3390/ani15121759 (PMC12189427; doi:10.3390/ani15121759)

Figure S1

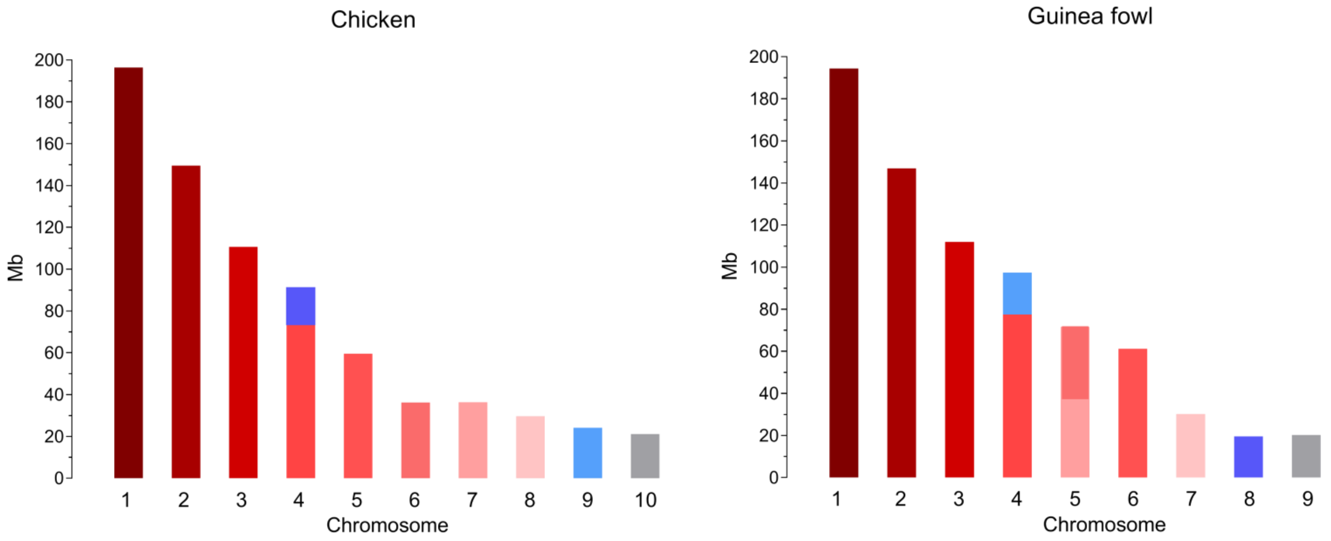

Figure S1. Comparative ideograms of chicken and guinea fowl 10 largest chromosomes. The colors reflect the chromosome rearrangements and their identity at sequence level determined by molecular cytogenetics and genome sequencing. The short arms of chromosome 4 result from translocations of different microchromosomes in each species (shown in light and dark blue). Based on data from Shibusawa et al 2002 and Vignal et al 2019)

Table S1

Chicken			Guinea fowl		
chr	***Mb***	***# intervals***	***chr***	***Mb***	***# intervals***
1	196.45	79	***1***	194.4	78
2	149.54	60	***2***	147.1	59
3	110.64	44	***3***	112	45
4	90.86	36	***4***	97.5	39
5	59.51	24	***5***	71.6	29
6	36.22	14	***6***	61.2	24
7	36.38	15			

Table S1. Number of 2.5 Mb intervals in the macrochromosomes of the chicken and the guinea fowl compared in the present analysis. The chromosome size in Mb is from the assembled genomes of each species at https://www.ncbi.nlm.nih.gov/genome/. NME 6 correspond to GGA5; GGA 6 and 7 correspond to NME 6.

Table S2

	Chicken									
Chr	1		2		3		4		1 - 4	
	r	p	r	p	r	p	r	p	r	p
GC	0.32	0.005	0.46	0.0002	0.35	0.0313	0.65	<0.0001	0.31	<0,0001
CGIs	0.15	0.187	0.30	0.018	0.16	0.3307	0.56	0.0004	0.26	0.0001
Genes	0.17	0.1447	0.27	0.0352	0.28	0.0816	0.51	0.0016	0.27	<0.0001

	Guinea fowl									
Chr	1		2		3		4		1 - 4	
	r	p	r	p	r	p	r	p	r	p
GC	0.13	0.3444	0.47	0.0018	0.29	0.1452	0.40	0.1118	0.31	0.0002
CGIs	0.07	0.6202	0.13	0.4303	0.09	0.6473	0.47	0.0571	0.19	0.024
Genes	0.01	0.9179	0.08	0.6292	-0.06	0.7584	0.33	0.1886	0.16	0.0605

Supplementary Table 2. Association between sequence parameters and recombination rates in individual chromosomes and grouped chromosomes (1 to 4). The associations are mainly non-significant in the guinea fowl compared to the chicken. The shaded cells show the significant correlation coefficients (Spearman´s r). p = P values for correlation tests.

Figures S2, S3 and S4 show percent of GC content , CpG islands and gene density in 2.5 Mb intervals along the chicken and guinea fowl macrochromosomes, respectively. See “*Conversion of physical distances along chromosomes into genomic distances”* in the main text for details in the construction of the frequency plots.

Figure S2. GC content

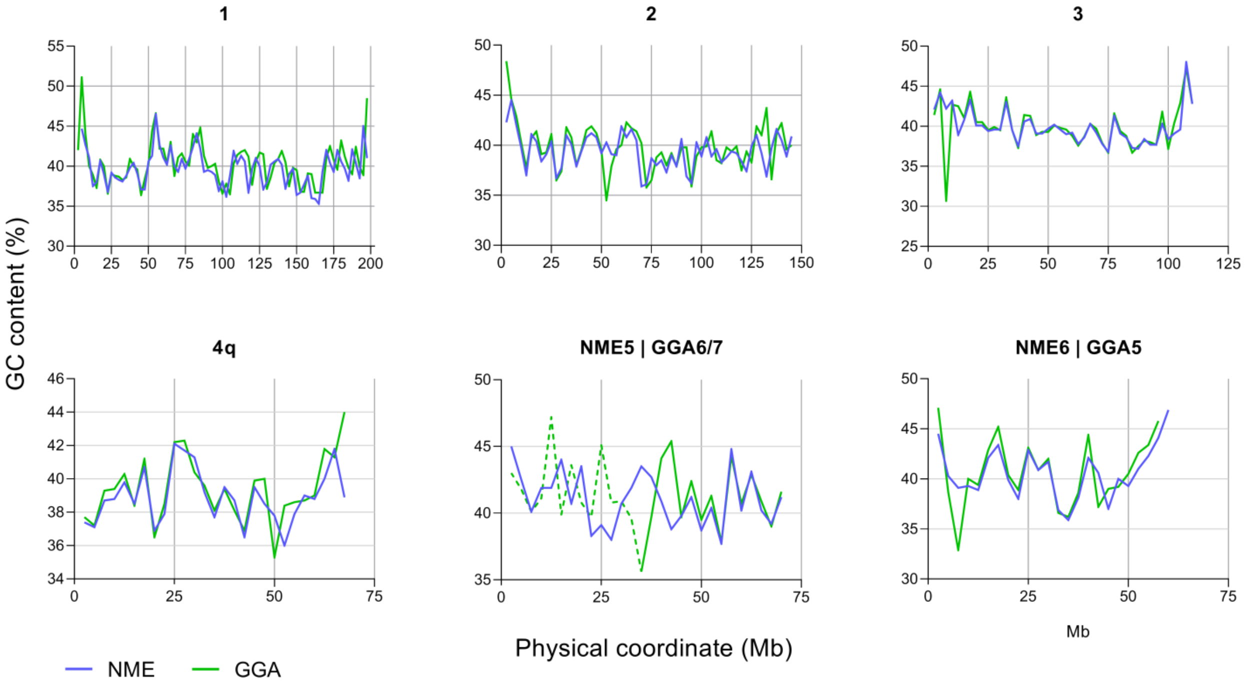

Figure S3. CGIs

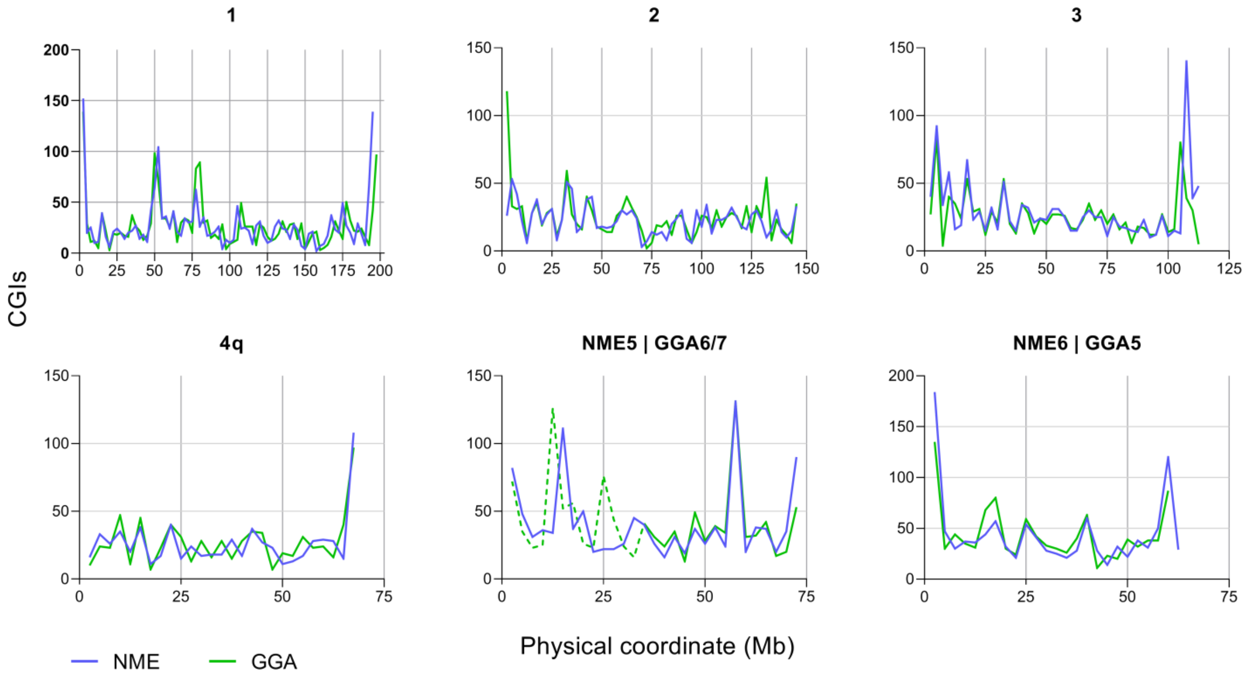

Figure S4. Gene density

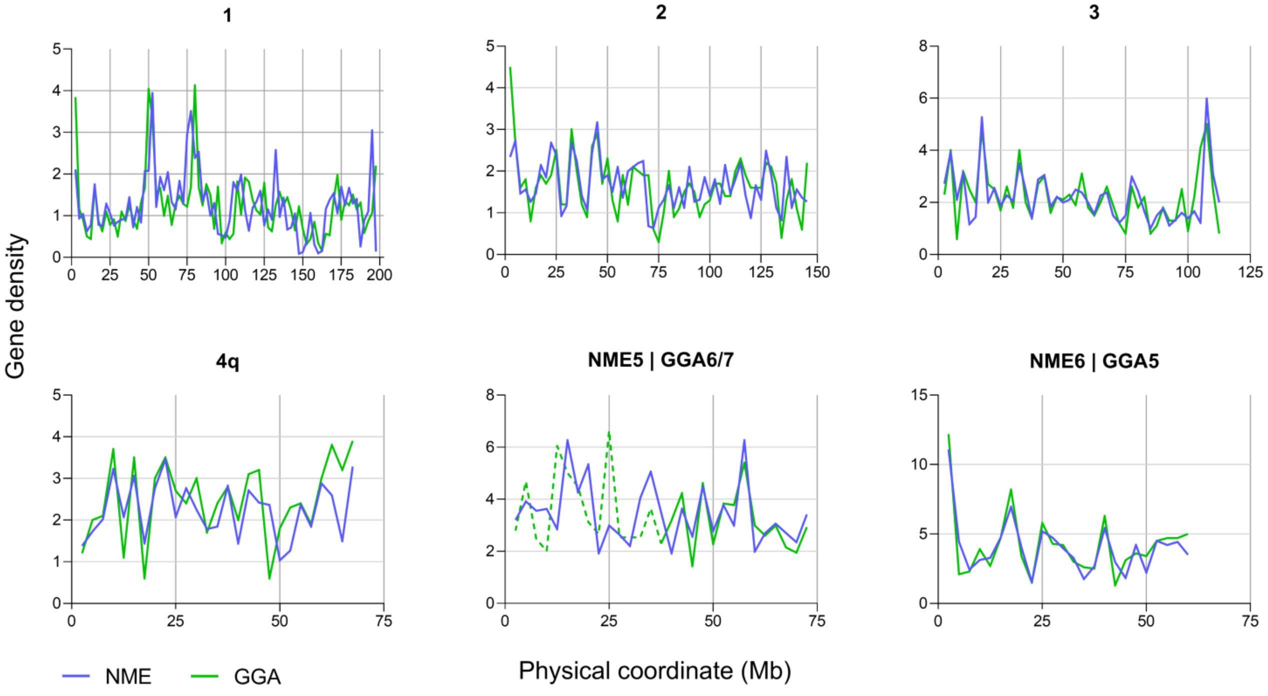

Supplement: Supplementary file 1 [file animals-15-01759-s001.zip › animals-3674454-supplementary.pdf]
